# Pre-dive Whole-Body Vibration Better Reduces Decompression-Induced Vascular Gas Emboli than Oxygenation or a Combination of Both

**DOI:** 10.3389/fphys.2016.00586

**Published:** 2016-11-30

**Authors:** Costantino Balestra, Sigrid Theunissen, Virginie Papadopoulou, Cedric Le Mener, Peter Germonpré, François Guerrero, Pierre Lafère

**Affiliations:** ^1^Environmental, Occupational, Ageing (Integrative) Physiology Laboratory, Haute Ecole Bruxelles-Brabant - HE2BBrussels, Belgium; ^2^DAN Europe Research DivisionRoseto, Italy; ^3^DAN Europe Research DivisionBrussels, Belgium; ^4^Anatomical Research and Clinical Studies (ARCS), Vrije Universiteit BrusselBrussels, Belgium; ^5^Anatomical Research Training and Education (ARTE), Vrije Universiteit BrusselBrussels, Belgium; ^6^Motor Sciences, Université Libre de BruxellesBrussels, Belgium; ^7^Dayton Lab, Department of Biomedical Engineering, University of North CarolinaChapel Hill, NC, USA; ^8^Center for Hyperbaric Oxygen Therapy, Military Hospital “Queen Astrid”Brussels, Belgium; ^9^ORPHY Laboratory, EA 4324, Université de Bretagne OccidentaleBrest, France

**Keywords:** decompression sickness/^*^etiology/metabolism, diving/^*^adverse effects, risk assessment, risk factors, preconditioning

## Abstract

**Purpose:** Since non-provocative dive profiles are no guarantor of protection against decompression sickness, novel means including pre-dive “preconditioning” interventions, are proposed for its prevention. This study investigated and compared the effect of pre-dive oxygenation, pre-dive whole body vibration or a combination of both on post-dive bubble formation.

**Methods:** Six healthy volunteers performed 6 no-decompression dives each, to a depth of 33 mfw for 20 min (3 control dives without preconditioning and 1 of each preconditioning protocol) with a minimum interval of 1 week between each dive. Post-dive bubbles were counted in the precordium by two-dimensional echocardiography, 30 and 90 min after the dive, with and without knee flexing. Each diver served as his own control.

**Results:** Vascular gas emboli (VGE) were systematically observed before and after knee flexing at each post-dive measurement. Compared to the control dives, we observed a decrease in VGE count of 23.8 ± 7.4% after oxygen breathing (*p* < 0.05), 84.1 ± 5.6% after vibration (*p* < 0.001), and 55.1 ± 9.6% after vibration combined with oxygen (*p* < 0.001). The difference between all preconditioning methods was statistically significant.

**Conclusions:** The precise mechanism that induces the decrease in post-dive VGE and thus makes the diver more resistant to decompression stress is still not known. However, it seems that a pre-dive mechanical reduction of existing gas nuclei might best explain the beneficial effects of this strategy. The apparent non-synergic effect of oxygen and vibration has probably to be understood because of different mechanisms involved.

## Introduction

Scuba diving is a sport with exhilaration, beauty, and fascination. This is probably why there are an estimated 10 million active recreational scuba divers worldwide (Trout et al., 2015). Additionally, diving is also key for environmental and scientific monitoring, construction and maintenance work, offshore oil exploitation, forensic, rescue, military, and filming purposes. However, the risks involved are often not advertised. Indeed, the overall rate of diving-related injury is 3.02 per 100 dives (Ranapurwala et al., 2014).

One of these injuries is decompression illness (DCI), a pathology affecting divers, astronauts, pilots and compressed air workers. Although DCI occurrence is relatively rare, with rates of 0.01–0.1% per dive (the higher end of the spectrum reflecting rates for commercial diving and the lower rates for scientific and recreational diving), the consequences can be dramatic (Ladd et al., 2002; Vann, 2004; Buzzacott, 2012). Indeed, DCI severity can vary from skin itching and marbled appearance to excruciating pain, convulsions, paralysis, coma and death. Over 60% of symptoms present in the first 3 h post dive, with some presenting as late as 48 h post-dive (Levett and Millar, 2008), and can be localized (joint pain in a particular articulation) or involve multiple systems. Therefore, divers should understand their limitations and how to prevent adverse outcomes.

Except for the more dramatic instance of arterial gas embolism by pulmonary overpressure, DCI is caused by bubble formation from dissolved inert gas in the tissues during decompression, vascular gas emboli (VGE). VGE can cause problems through direct mechanical effects (by blocking or distorting blood vessels) but also from the associated inflammatory response they trigger (Blatteau et al., 2014). Therefore, since decompression sickness (DCS) risk is inherently dependent on the dive profile and most importantly on the ascent profile (Marroni et al., 2004), it is managed by adhering to decompression schedules dictated by tables or dive computers, which are based on a decompression model or algorithm. Current algorithms include multi-tissue, diffusion, split phase gradient, linear-exponential, asymmetric tissue, thermodynamic, varying permeability, reduced gradient bubble, tissue bubble diffusion, and linear-exponential phase models. All of these models aim to limit bubble formation and growth during the decompression phase. Indeed, if VGE load is high, so is the risk of DCI. Moreover, VGE can cross from the central venous to the arterial circulation via a pulmonary shunt or a Patent Foramen Ovale. The more VGE are present after the dive, the higher this risk (Nishi, 1972; Blogg et al., 2014; Pollock and Nishi, 2014). However, all models can only partly describe reality and are by essence incomplete; they merely serve to “organize our ignorance” of the phenomenon. Therefore, the past 15 years or so, have witnessed changes and additions to diving protocols and table procedures, such as shorter nonstop time limits, slower ascent rates, shallow safety stops, ascending repetitive profiles, deep decompression stops, helium-based breathing mixtures, permissible reverse profiles, multilevel techniques, both faster, and slower controlling repetitive tissue halftimes, smaller critical tensions, longer flying-after-diving surface intervals, and others (Wienke, 2009). Nonetheless, VGE are still known to form in the body after many dives, even those done well within the limits of the accepted decompression model. Understanding these processes physiologically has been a challenge for decades and there are a number of questions still unanswered such as the exact primer for bubble formation, theories to account for micronuclei stability (hydrophobicity of surfaces or tissue elasticity), or the relevance of predisposing factors (dehydration for instance) (Papadopoulou et al., 2013).

Since non-provocative dive profiles are no guarantor of protection against DCS, novel means are required for its prevention including pre-dive procedures that could induce more resistance to decompression stress. This idea has prompted a change in research paradigm. Indeed, since several years, field research focuses on “preconditioning” methods that might attenuate bubble formation post-dive. Several practical, simple and feasible pre-dive measures have been studied such as endurance exercise (Blatteau et al., 2005; Castagna et al., 2011), pre-dive exposition to a warm environment (Blatteau et al., 2008), oral hydration (Gempp et al., 2009) or ingestion of dark chocolate (Theunissen et al., 2015). Others have tested the benefit of pre-dive oxygenation (Castagna et al., 2009; Bosco et al., 2010), or whole-body vibration (Germonpré et al., 2009). All of these studies show a positive effect with a significant decrease of post-dive VGE. Several hypotheses have been advocated to explain the possible protective effect: rheological changes affecting tissue perfusion, endothelial adaptation with nitric oxide pathway, up-regulation of cytoprotective proteins, and reduction of pre-existing gas nuclei from which bubbles originate (Gempp and Blatteau, 2010).

From a physiological point of view, comparing the effectiveness of preconditioning is interesting because it would allow to identify critical factors in the physiopathology of DCS. Similarly, it would be of practical interest to examine the possible synergy or antagonism of preconditioning methods when combined. Therefore, the aim of this study is to compare the effect on post-dive VGE of different types and combination of preconditioning methods: oxygenation, whole-body vibration and vibration associated with oxygenation.

## Materials and methods

All experimental procedures were conducted in accordance with the Declaration of Helsinki (World Medical Association, 2013) and were approved by the Academic Ethical Committee of Brussels (B200-2009-039). All methods and potential risks were explained in detail to the participants.

### Study population

After written informed consent, 6 healthy male divers (Minimum certification “Autonomous Diver” according to European Norm EN 14153-2 or ISO 24801-2, with at least 50 logged dives) volunteered for this study. They were selected from a large sports diver population in order to obtain a group of comparable age [30–40 years, 34.8 ± 5.3 (mean ± SD)], body composition (BMI between 20 and 25, 23.7 ± 1.1) and comparable health status: non-smokers with regular but not excessive physical activity (aerobic exercise one to three times a week). The divers that participated in this protocol have already participated in some of our experiments and are known as being consistent “bubblers” (Theunissen et al., 2015). Prior to entry into the study, they were assessed fit to dive. None of the subjects had a previous history of decompression sickness and none of them were on any cardio-active medication.

Participants were instructed not to dive 72 h prior to the experimental dive. They were also were asked to refrain from strenuous exercise and nitrate-rich food for 48 h before the tests (Blatteau et al., 2005).

### Dive protocol

Each diver performed 6 standardized dives with a minimum interval of 1 week between then. This standard dive profile (see below) was performed at least three times under normal conditions i.e., without preconditioning (“control”), and several times under experimental conditions, when the effects of several methods of preconditioning were measured, making each diver his own control. The order of the experimental dives was randomized. Preconditioned dives were preceded either by a 100% normobaric oxygen breathing session (O_2_) through a non-rebreather facemask (Teleflex medical BVBA, Vianen, Nederland), a whole-body vibration session (Vib) using a commercially available vibration mattress (VM 9100 RM, HHP Products, Karlsruhe, Germany), or a combination of both simultaneously (VibO_2_). Vibration frequencies ranged from 35 to 40 Hz along the whole body thanks to 11 motors embedded in the mattress. The subject lay motionless on the mattress during the entire vibration session, or a non-vibrating mattress while only breathing oxygen. All preconditioning had duration of 30 min and ended 1 h before the start of the dive.

Dives were performed to a depth of 33 mfw (0.4 MPa) for 20 min in a pool environment (Nemo33, Brussels, Belgium) with a water and air temperature of 33 and 29°C respectively, thus needing no thermal protection suit.

The descent was done at 20 m.min^−1^. At depth, subjects were asked to swim slowly without effort, then came back to the surface with an ascent speed of 10 m.min^−1^. Since this depth-time profile falls within accepted “no-decompression limits” (NAVSEA, 2008), no decompression stop was added to the profile.

Participant safety was guaranteed through the buddy system, a procedure in which two divers, “the buddies,” operate together as a single unit so that they are able to monitor and help each other. Moreover, a safety diver was ready to intervene at 20 m depth.

### Measurements

Bubbles which form as a consequence of decompression can be detected as VGE by ultrasonic methods (Møllerløkken et al., 2016). Using a two-dimensional echocardiography technique (Vivid 7, GE Healthcare, Pollards Wood, UK), a frame-based counting method as described by Germonpré et al. (2014) was used to quantify VGE.

In this method, in the left lateral supine position, a cardiac four-chamber view is obtained by placing the probe at the level of the left fifth intercostal space. It is necessary to modify the standard four-chamber view by rotating the probe slightly ventrally (in the direction of the xyphoid process) so the right atrium and ventricle can be fully visualized. A series of at least 15 cardiac cycles are recorded while keeping the probe immobile. Each diver was evaluated at three time points: before the dive, at 30 min and at 90 min after surfacing. They were made at the end of a period during which subjects remain at rest (without flexion) and following active provocation by two deep knee bends (with flexion). In total, 5 videos of 15 cardiac cycles were recorded for each dive.

At a later stage, these recordings are reviewed using the MPEGVue software (GE Healthcare, Pollards Wood, UK). First, the pre-dive echography loops are reviewed in order to identify intra-cardiac structures that may mimic VGE (e.g., papillary muscles, valve leaflets, Chiari network, Valsalva sinus). Then, the post-dive echography is reviewed and played in a loop at real-time speed in order to rapidly assess the presence or not of circulating bubbles.

In cases where bubbles are seen, a formal bubble counting procedure is performed. Using the pause button, the loop is frozen at the start, and then with the forwards and backwards buttons, an image frame is selected in end-diastolic/proto-systolic position and bubbles are counted in both the right atrium and ventricle. Ten consecutive frames are analyzed and the bubble count is averaged over these 10 frames.

**Figure 1:**
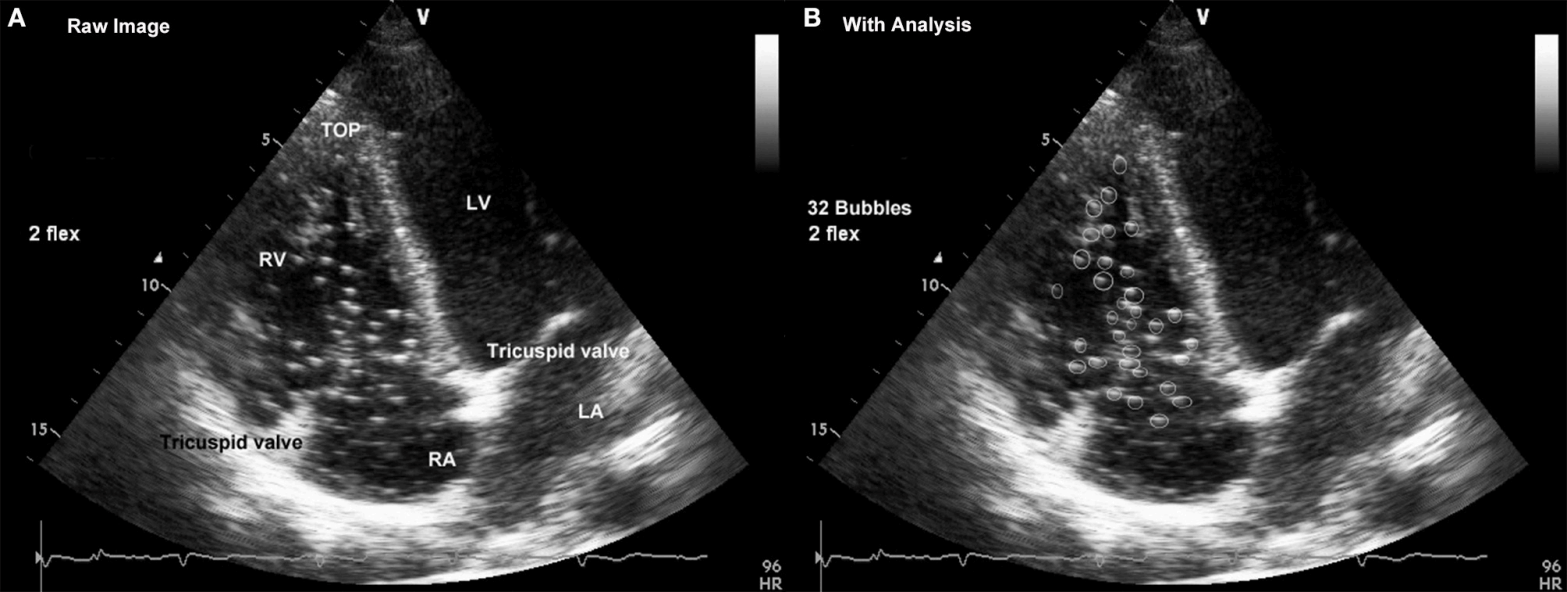
**Cardiac four-chamber view obtained by placing the probe at the level of the left fifth intercostal space. (A)** Raw Image: Landmark structures in the right heart are easily identified with the “top” of the right ventricle (RV) and the tricuspid annulus on either side of the right atrium that constitute the “upper” border of the right atrium (RA). **(B)** With Analysis: Bubble signals are identified as bright spots and counted individually; tricuspid valve leaflets and other fixed structures (e.g., papillary muscles in the top of the right ventricle) are not counted. (LA: Left Atrium; LV: Left Ventricle).

The counting was performed independently twice by two trained scientists acquainted with the method used (CB, PG) the numbers of VGE considered for calculation were those that reached consensus (Figure 1).

### Statistical analysis

Since all data passed the Kolmogorov-Smirnov and Shapiro-Wilk tests, allowing us to assume a Gaussian distribution, they were analyzed with repeated-measures ANOVA with Bonferroni *post-hoc* test.

Taking the mean bubble count of the control dives (without preconditioning) as 100%, percentage changes were calculated for each preconditioning protocol, allowing an appreciation of the magnitude of change rather than the absolute values.

All statistical tests were performed using a standard computer statistical package, GraphPad Prism version 5.00 for Windows (GraphPad Software, San Diego California USA). A threshold of *P* < 0.05 was considered statistically significant. All data are presented as mean ± standard error on mean (SEM).

## Results

None of the dives resulted in decompression sickness symptoms.

**Figure 2:**
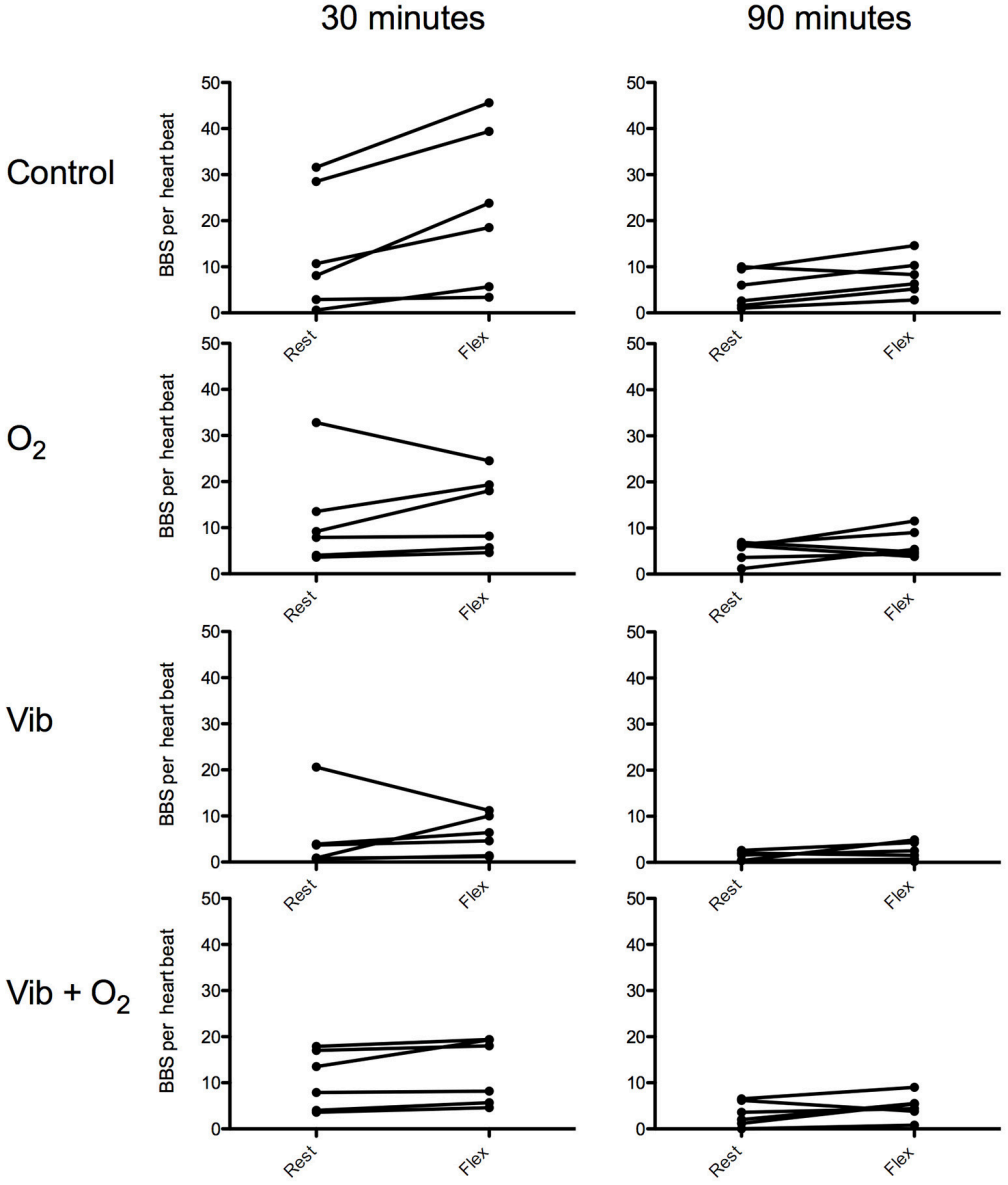
**Individual VGE count after a 33 mfw dive without (control) and with preconditioning for 30 min ending 1 h before the dive [oxygenation (O_2_), whole body vibration (Vib) or a combination of both (Vib+O_2_)]**. VGE counts are shown at 30 and 90 min post-dive, before and after knee flex.

After the control dives, the absolute VGE counts ranged from 15.2 ± 4.3 VGE per cardiac cycle at rest to 18.9 ± 4.3 after knee flexion. After the experimental dives, absolute maximal bubble counts were 4.9 ± 3.2 and 5.6 ± 1.9 (Vib); 8.1 ± 2.9 and 9.8 ± 2.9 (Vib+O_2_) and 10.9 ± 4.9 VGE and 14.4 ± 2.3 (O_2_), VGE per cardiac cycle respectively without and with knee flexion. Maximal bubble counts were systematically used to evaluate the magnitude of the change independently of the bubble peak kinetics. However, it can be seen on Figure 2 that it was preferentially obtained after active provocation (knee flexion) at the 30 min post-dive measurement.

**Figure 3:**
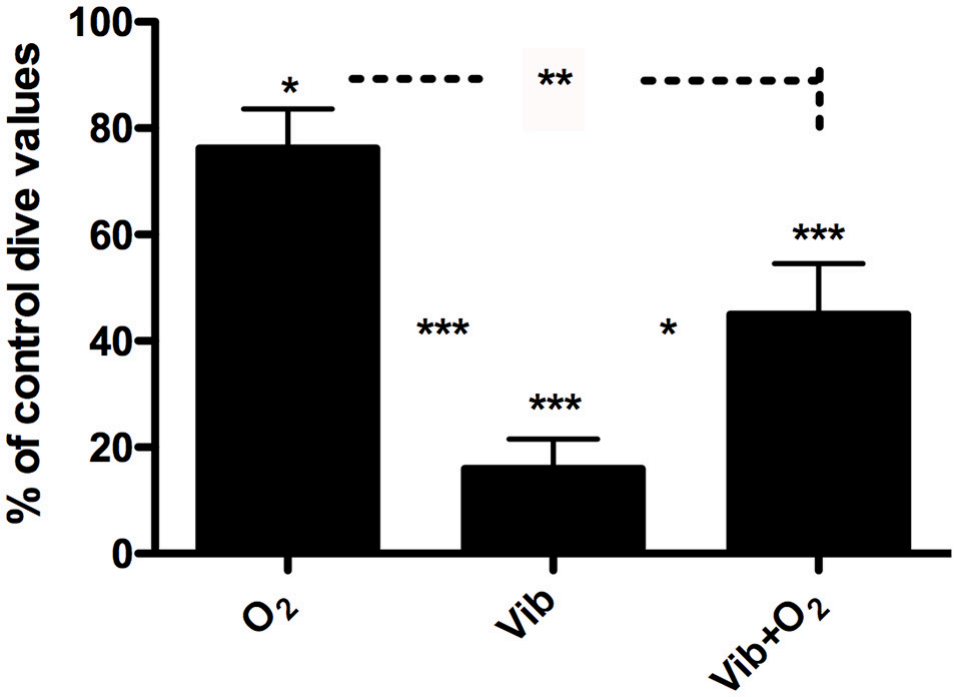
**Percentage variation of post-dive VGE count (mean ± SEM) after a 20 min dive to 33 mfw after different preconditioning measures: oxygen prebreathing (O_2_), whole-body vibration (Vib), or a combination of both (Vib+O_2_)**. Control dive value is taken as 100%. Each subject is compared to his own control dive value. (^***^p < 0.001; ^**^p < 0.01; ^*^p < 0.05) (*n* = 6).

The effect of the different preconditioning protocols on bubble formation after the dive is illustrated in Figure 3. Each pre-dive procedure induced a significant post-dive VGE change (ANOVA, *p* < 0.0001, df = 23). Compared to baseline, this variation is characterized by a decrease in VGE count of 23.8 ± 7.4% after oxygen breathing (O_2_, *p* < 0.05), 84.1 ± 5.6% after vibration (Vib, *p* < 0.001), and 55.1 ± 9.6% after vibration combined with oxygen (Vib+O_2_, *p* < 0.001). The effectiveness of the various protocols was also statistically significantly different when they were compared to each other, whole-body vibration (Vib) being the most effective (*p* < 0.001).

## Discussion

The origin and formation of VGE is still incompletely understood. Bubble formation after hyperbaric exposure is not simply the consequence of inert gas supersaturation during decompression. Experimental data have shown that supersaturation by itself will not produce bubbles in a homogenous fluid unless the pressure reduction is about 1400 ATA for air (Zheng et al., 1991)! These “homogenous nucleation” limits have been studied for several gases and may vary according to their solubility: 120 ATA for methane, 190 ATA for nitrogen and 350 ATA for helium (Finkelstein and Tamir, 1985). It seems thus that homogenous nucleation cannot be held responsible for (diving decompression) VGE generation. Indeed, numerous experiments indicate that bubbles originate as pre-existing gas nuclei (Yount et al., 1979; Vann et al., 1980; Christman et al., 1986; Lee et al., 1993). Nitrogen, diffusing out of the tissues during decompression, would preferably fill these gas nuclei rather than transfer as molecular nitrogen to blood. This causes the gas nuclei to grow and spill out nitrogen gas bubble into the bloodstream. Here, they either grow or shrink depending on surface tension and free gas tension (Blatteau et al., 2006). Although we only have indirect evidence of the presence of these gas nuclei, if this hypothesis is correct, eliminating gas nuclei before the dive would result in lower bubble production after the dive.

The aim of our study was to compare the effect of three different preconditioning methods on post-dive VGE. All three methods have already been shown to significantly decrease the number of VGE compared to controls dives in a way that is consistent with data in the literature (Castagna et al., 2009; Germonpré et al., 2009). The observation of a bubble peak after active provocation (knee flexing) at the 30 min post-dive measurement is also coherent with the literature (Blogg and Gennser, 2011).

The role of oxygen breathing (O_2_) in the reduction of DCS risk has been extensively investigated before altitude decompression (Webb and Pilmanis, 2011; Foster et al., 2013; Webb et al., 2016). Several studies have shown that a single hyperbaric oxygen exposure before diving appeared to be beneficial for preventing the occurrence of DCS in animals (Butler et al., 2006; Arieli et al., 2007; Katsenelson et al., 2007) and reducing bubble generation in humans (Landolfi et al., 2006). This approach did not seem as effective when normobaric oxygen was used as pretreatment before a simulated dive in rats (Butler et al., 2006). Nonetheless, Castagna et al. found that oxygen prebreathing provides a significant reduction in decompression-induced bubble formation, regardless of the experimental conditions (Castagna et al., 2009). This is confirmed by the work of Bosco et al. although hyperbaric oxygen seems more effective (Bosco et al., 2010).

Denitrogenation *per-se* does not seem preponderant in the effectiveness of oxygen prebreathing (Gempp and Blatteau, 2010). On the contrary, the proposed mechanism is based on the ability of oxygen to replace nitrogen in the gas nuclei by diffusion. Reduction of tissue oxygen pressure after switching from oxygen to air would then enhance the consumption of oxygen from the gas nuclei, thus eliminating it completely (Arieli et al., 2002). Another possibility is that oxygen administration induces prolonged hemodynamic effects such as decrease in heart rate, cardiac output, and increase in systemic vascular resistance (Waring et al., 2003; Thomson et al., 2006), leading to a reduction in inert gas load in peripheral tissues during diving—which would subsequently reduce post-dive inert gas bubble formation.

Whilst exposure to vibration is traditionally regarded as perilous, recent research has focused on potential benefits. Indeed, it was demonstrated that 30 min of whole-body vibration before a wet dive had preventive effects on post-dive VGE (Germonpré et al., 2009).

Vibration is a mechanical oscillation, i.e., a periodic alteration of force, acceleration and displacement over time. Vibration exercise, in a physical sense, is a forced oscillation, where energy is transferred from an actuator (i.e., the vibration device) to a resonator (i.e., the human body). As a consequence acute physiological responses can be observed.

As in this study there was no observed change in FMD after vibration, the authors did not believe an NO mediated mechanism was involved; rather, a mechanical dislodgement of VGE precursors (located in microcrevices between endothelial cells). This is illustrated by the prompt increase of post-dive VGE after a few seconds of exposure on the vibration mattress (Germonpré et al., 2009) or by the knee flexion maneuver, which by increasing the shear stress on the vessel wall, increases the liberation of any existing, adherent gas bubbles (Møllerløkken et al., 2016). Another regional effect may be involved. Vibrations applied to a limb have been shown to increase the rate of lymphatic drainage. Since evacuation of gas bubbles (and nuclei) is possibly happening in part by means of lymphatic fluid (Leduc et al., 1981; Madhavan et al., 2006), it cannot be excluded that an increased lymphatic flow during the whole-body vibration session is partly responsible for the observed effect. Indeed, vibrations could induce, by force transmission, a modification of endothelial spatial conformation. Secondly, the increase in lymphatic circulation induced by vibration (Leduc et al., 1981; Balestra, 2014) would allow the elimination of inter-cellular tissue-located micronuclei.

Therefore, although the exact mechanism by which gas nuclei are eliminated from the vessel by mechanical vibration remains to be clarified, the benefits of whole-body vibration (Vib) are best explained by the mechanical action of vibration on endovascular and tissue localization of the micronuclei.

As stated above, bubbles either grow or shrink depending on surface tension and free gas tension. This also applies to gas nuclei (Papadopoulou et al., 2013), which are thought to be stabilized by trapping in intercellular cervices (Tikuisis, 1986) or by coating with surface-active molecules like surfactant, platelets or proteins (Letho and Laitinen, 1979; Thorsen et al., 1987, 1993). It is thus reasonable to assume a synergistic effect of oxygen and vibration in VibO_2_ preconditioning. Yet it is the opposite that we observed, Vib+O_2_ being superior to O_2_ but inferior to Vib in decreasing post-dive VGE counts.

This absence of synergy could be explained by the fact that the two modes of preconditioning, mechanical or diffusion, could act on the same nuclei and thus be in direct competition.

Oxygen breathing will produce a higher level of reactive oxygen species (ROS), which lead to oxidative stress. Increased production of ROS favors vascular dysfunction (Kuznetsova et al., 2014), inducing a decrease in endovascular NO production, altered vascular permeability and inflammation, accompanied by the loss of vascular modulatory function, the imbalance between vasorelaxation and vasoconstriction, and the aberrant expression of inflammatory adhesion molecules (Bielli et al., 2015). All these mechanisms could then counteract the influence of vibration. However, in the study by Germonpre et al. vibration sessions did not result in a significant modification of endothelial reactivity, as indicated by the FMD measurements (Germonpré et al., 2009). Therefore, the probability that NO production or vessel wall reactivity was significantly altered in the present study (that uses the same experimental setting) is low. It is also possible that since oxygen administration induced prolonged hemodynamic effects such as decrease in heart rate, cardiac output, and increase in systemic vascular resistance (Waring et al., 2003; Thomson et al., 2006), blood flow was sufficiently reduced to diminish the necessary shear forces induced by vibration reducing the capacity of the mechanical intervention to dislodge gas nuclei.

However, the most likely hypothesis explaining this lack of potentiation is that mechanical denucleation is the preponderant mechanism.

## Conclusions

The precise mechanism that induces the decrease in post-dive VGE and thus makes the diver more resistant to decompression stress is still not known. Different preconditioning methods based on oxygen prebreathing or whole-body vibration produce significant results. However, it seems that a pre-dive mechanical reduction of existing gas nuclei might best explain the beneficial effects observed. The apparent non-synergic effect of oxygen and vibration has probably to be understood because of different mechanisms involved.

## Author contributions

All authors gave substantial contributions to the conception or design of the work; the acquisition, analysis, and interpretation of data for the work. PG, CB, FG, and PL were responsible for drafting the work or revising it critically for important intellectual content. Final approval of the version to be published was made by CB, FG, and PL.

## Funding

This study is part of the Phypode Project, financed by the European Union under a Marie Curie Initial Training Network Program FRP/2007-2013/ under REA grant agreement n° 264816.

### Conflict of interest statement

The authors declare that the research was conducted in the absence of any commercial or financial relationships that could be construed as a potential conflict of interest.
